# An Initial Evaluation of Human Plasma cMLC-1: A Potential Protein Biomarker for Trastuzumab-Induced Cardiotoxicity, Breast Cancer Screening and Progression

**DOI:** 10.3389/fonc.2022.809715

**Published:** 2022-05-03

**Authors:** Ling Yu, Read Allen, Lin Jia, Ting Sun, Steven J. Isakoff, Marielle Scherrer-Crosbie, Allison M. Kehlmann, Hui Zheng, Amy Ly, Charlotte S. Walmsley, Katherine Hesler, Ava N. Varasteh, Christopher J. Pinto, Daniel E. McLoughlin, Wenjin Wu, Xinhui Wang

**Affiliations:** ^1^ Division of Gastrointestinal and Oncologic Surgery, Department of Surgery, Massachusetts General Hospital, Harvard Medical School, Boston, MA, United States; ^2^ Key Laboratory of Luminescence Analysis and Molecular Sensing, Ministry of Education, School of Materials and Energy, Southwest University, Chongqing, China; ^3^ Termeer Center for Targeted Therapies, Massachusetts General Hospital Cancer Center, Boston, MA, United States; ^4^ Perelman Center for Advanced Medicine, Cardiovascular Medicine Division, The Hospital of the University of Pennsylvania, Philadelphia, PA, United States; ^5^ Biostatistics Center, Massachusetts General Hospital, Harvard Medical School, Boston, MA, United States; ^6^ Department of Pathology, Massachusetts General Hospital, Harvard Medical School, Boston, MA, United States; ^7^ Division of Monoclonal Antibodies, Office of Biotechnology Products, Office of Pharmaceutical Science, Center for Drug Evaluation and Research, U.S. Food and Drug Administration, Bethesda, MD, United States

**Keywords:** cardiac myosin light chain 1(cMLC-1), biomarkers, trastuzumab-induced cardiotoxicity, breast cancer screening, breast cancer progression

## Abstract

**Background:**

Trastuzumab is a targeted therapy for human epidermal growth factor receptor 2 (HER2)-positive breast cancer. However, trastuzumab-induced cardiotoxicity (TIC) has been reported when trastuzumab is administered to patients as a single agent or combined with anthracycline. Currently no means for detecting the early onset of TIC such as a protein biomarker is available. In this regard and based on promising results from a preliminary animal study, the potential of cardiac myosin light chain 1(cMLC-1) as a biomarker to predict TIC, screen patients for breast cancer and monitor tumor progression in breast cancer patients was evaluated.

**Methods:**

Archived plasma samples collected before and after trastuzumab treatment at various fixed time points from 15 HER2^+^ patients with or without cardiotoxicity, recently collected plasma samples from 79 breast cancer patients (40 HER2^+^, 39 HER2^-^), and 46 healthy donors were analyzed for cMLC-1 levels using an enzyme-linked immunosorbent assay (ELISA).

**Results:**

An elevated plasma cMLC-1 level was found to be associated with TIC in 3 out of 7 (43%) trastuzumab-treated HER2^+^ breast cancer patients. However, this study provided an opportunity for us to study plasma cMCL-1 levels in breast cancer patients. It was demonstrated that elevated plasma cMCL-1 is associated with breast cancer. The cutoff cMLC-1 concentration is estimated to be 44.99 ng/mL with a sensitivity of 59.49% (95%CI: 48.47%-69.63%) and specificity of 71.74% (95%CI: 57.45% -82.68%). We also found a noticeable but not significantly more elevated plasma cMCL-1 level in HER2^-^ than in HER2^+^ breast cancer patients with the given sample sizes. As a result, improved sensitivity of 79.49% (95%CI: 64.47%-89.22%) with the specificity of 63.04% (95%CI:48.60%-75.48%) were obtained for cMLC-1 to predict HER2^-^ breast cancer with the cutoff at 37.17 ng/mL. Moreover, this study determined that cMLC-1 level was significantly higher in patients with metastatic breast cancer than in patients with non-metastatic breast cancer.

**Conclusions:**

While the analysis of cMLC-1 levels in the plasma of a limited number of trastuzumab-treated HER2^+^ breast cancer patients failed to fully support its identification as a blood protein biomarker for predicting TIC, additional analyses of plasma cMLC-1 levels did significantly establish its correlations with breast cancer and disease progression. Our findings shed light on and filled, to some extent, the gap of knowledge of the potential of cMLC-1 as a blood protein biomarker for screening breast cancer and monitoring disease progression of breast cancer.

## Background

Breast cancer (BC) is one of the most common malignancies in the United States, with over 280,000 new cases expected in 2021 (1). Approximately one in five women diagnosed with breast cancer worldwide will have an aggressive form of the disease with human epidermal growth factor receptor 2 (HER2) gene amplification or protein overexpression, known as HER2^+^ subtype (2). Trastuzumab (Herceptin^®^) is a humanized monoclonal antibody specifically targeting HER2 that is used to treat both early- and late-stage HER2^+^ breast cancer. When started before or after surgery to treat early disease, the drug is administered every 21 days for a total of one year. For advanced breast cancer, treatment is typically given as long as the patient continues to derive clinical benefit (3). Trastuzumab is typically prescribed as a single agent or in combination with standard chemotherapy regimens such as anthracyclines. However, trastuzumab treatment is associated with cardiac dysfunction, which manifests as a decrease in left ventricular ejection fraction (LVEF) and heart failure (4–6). Trastuzumab-induced cardiotoxicity (TIC) has been reported to occur in up to 7% of patients when trastuzumab was used as a single agent (7). When combined trastuzumab with an anthracycline, however, cardiotoxicity increases dramatically to up to 27% of patients (7).

Alarmingly, a decrease in LVEF has even been detected in asymptomatic breast cancer patients administered trastuzumab. Early identification of breast cancer patients for left ventricular dysfunction following trastuzumab therapy is essential for early initiation of cardioprotective measures. A blood-based biomarker for TIC would be better as an ongoing surveillance strategy than the current system of echocardiographic LVEF measurement to reveal TIC. However, to date, no such biomarker has been identified and validated for clinical use. Previous studies focused mainly on evaluating the potential of troponins I and T, brain natriuretic peptide (BNP), N-terminal pro b-type natriuretic peptide (NT-proBNP) and high-sensitivity C-reactive protein (hs-CRP) as blood protein biomarkers to predict TIC. Sawaya et al. revealed an association between troponin I (TnI) (also known as cardiac troponin I) levels at 3 months post-treatment with trastuzumab and development of cardiotoxicity at 6 months (8). Later, Onitilo et al. reported that elevated hs-CRP, but not BNP or troponin I, predicted decreased LVEF with a sensitivity of 92.9% but with a specificity of only 45.7%. With such a high false positives rate, this assay does not reliably predict toxicity (9). Recently, Zardavas et al. found that baseline (before trastuzumab treatment) troponin I and T levels were elevated in 13.6% (56 of 412) and 24.8% (101 of 407) patients, respectively, and that these measurements were associated with a significantly increased risk of reduced LVEF (10). While these findings are encouraging, these efforts indicate that the search for better biomarkers for early prediction and identification of TIC must continue. Troponin I is considered as a sensitive and specific biomarker in the diagnosis of myocardial infarction. However, it is not sensitive and specific for the diagnosis of early stage of TIC (11). Cardiac myosin light chain-1 (cMLC-1, also known as myosin essential light chain (ELC)), is encoded by the MYL3 gene and is a part of the myosin complex that plays an important role in cardiac muscle contraction (11, 12). Impaired integrity of damaged or injured cardiomyocytes leads to release of cMLC-1 from the myocardium into circulation (13–15). Past studies have shown that circulating cMLC-1 protein was elevated in patients a few hours after acute myocardial infarction, and peaked on days 2 to 4 post infarction (16, 17). In addition, when serum levels of cMLC-1 and creatine kinase (CK) were measured in serial samples from 49 patients with acute myocardial infarction, the results suggested that serum cMLC-1 is a better marker than CK in predicting LVEF changes (18).Thus, we investigated the possibility of cMLC-1 as a potential biomarker for TIC in mice. Using echocardiography, we found that trastuzumab significantly reduced LVEF (11). Importantly, this reduced LVEF was associated with elevated levels of serum cMLC-1 in mice (11). The initial objective of this study was to evaluate for the first time the potential of cMLC-1 as a blood biomarker for TIC in breast cancer patients using plasma samples collected from HER2^+^ breast cancer patients, who had been treated with trastuzumab and either developed cardiotoxicity or did not. In turn, this effort led to analyses of cMLC-1 plasma levels as a prognostic indicator of breast cancer as well as disease progression in breast cancer patients.

## Materials And Methods

### Patients

Archived human plasma samples (n=15) were collected at multiple time points from a relatively homogenous patient population. The cohort consisted of women with newly diagnosed breast cancer administered anthracyclines followed by taxanes and trastuzumab. Approximately 50% of these patients (7/15) were selected because they had developed TIC and another ~50% of these patients (8/15) were selected because they had not developed TIC for this study. This experimental design did not involve in any way the incidence of TIC (**Tables 1**, **2**). Cardiotoxicity was defined using the Cardiac Review and Evaluation Committee for Trastuzumab (CREC) criteria as a decrease of more than 10% in the echocardiographic LVEF to a value of less than 55%. The women had been monitored every 3 months. The plasma samples had been collected and banked under the Massachusetts General Hospital Institutional Review Board (IRB protocol 2006P000886).

**Table 1:** Comparisons of plasma cMLC-1 levels at different time points in trastuzumab-treated BC patients.

Patient	Time	Mean	*p* value
#1	baseline *vs.* 3-month	55.05 ± 6.37 *vs.* 65.32 ± 2.72	0.230
#1	baseline *vs.* 6-month	55.05 ± 6.37 *vs.* 75.57 ± 7.07	0.010
#2	baseline *vs.* 3-month	17.60 ± 1.10 vs. 27.95 ± 3.38	0.050
#2	baseline *vs.* 6-month	17.60 ± 1.10 vs. 23.77 ± 4.02	0.140
#3	baseline *vs.* 3-month	0.57 ± 0.74 *vs.* 110.09 ± 26.94	0.001
#3	baseline *vs.* 6-month	0.57 ± 0.74 *vs.* 91.63 ± 15.36	0.002
#3	3-month *vs.* 6-month	110.09 ± 26.94 *vs.* 91.63 ± 15.36	0.264
#3	6-month *vs.* 9-month	91.63 ± 15.36 *vs.* 51.16 ± 0.64	0.059
#4	baseline *vs.* 6-month	18.19 ± 1.45 *vs.* 20.49 ± 2.02	0.076
#4	3-month *vs.* 6-month	12.96 ± 0.61 *vs.* 20.49 ± 2.02	0.035
#5	baseline *vs.* 3-month	26.57 ± 5.33 *vs.* 18.42 ± 1.81	0.148
#5	baseline *vs.* 6-month	26.57 ± 5.33 *vs.* 27.67 ± 1.13	0.410
#5	3-month *vs.* 6-month	18.42 ± 1.81 *vs.* 27.67 ± 1.13	0.024
#5	3-month *vs.* 9-month	18.42 ± 1.81 *vs.* 24.88 ± 1.41	0.053

**Table 2:** Comparisons of plasma cMLC-1 levels before and 3-month after trastuzumab treatment in BC patients.

Patient	Baseline	3-month	*p* value	Diagnosed Cardiotoxicity
#1	55.05 ± 6.37	65.32 ± 2.72	0.230	NO
#2	17.60 ± 1.10	27.95 ± 3.38	0.050	NO
#3	0.57 ± 0.74	110.09 ± 26.94	0.001	YES
#4	18.19 ± 1.45	12.96 ± 0.61	0.040	YES
#5	26.27 ± 5.33	18.42 ± 1.81	0.148	NO
#6	209.40 ± 31.11	298.70 ± 67.75	0.083	YES
#7	78.82 ± 3.65	42.38 ± 0.61	0.005	YES
#8	14.35 ± 0.11	119.40 ± 0.09	9.42E-07	NO
#9	33.24 ± 3.56	28.67 ± 0.25	0.164	YES
#10	0.22 ± 0.10	1.04 ± 0.75	0.194	NO
#11	89.48 ± 13.69	39.76 ± 2.82	0.035	NO
#12	72.18 ± 5.82	60.25 ± 6.88	0.158	NO
#13	48.96 ± 5.56	55.61 ± 8.05	0.283	NO
#14	100.10 ± 28.01	79.08 ± 19.40	0.217	YES
#15	60.54 ± 17.20	174.60 ± 49.23	0.018	YES

For the additional studies detailed in this report, patients with HER2^+^ (n=40) and HER2^-^ (n=39) breast cancer were recruited from the Massachusetts General Hospital Cancer Center between March 2018 and January 2020 (**Tables 3**, **4**). Based on existing clinical guidelines (19), HER2^+^ was defined as ≥2.0 amplification of HER2 by Fluorescent *In Situ* Hybridization (FISH) as noted on the pathology report from the date of original diagnosis, or as 2-3+ by immunohistochemistry (IHC) if FISH was not available. Patients who were receiving trastuzumab as standard therapy were also included in the HER2^+^ cohort, even if IHC and FISH did not meet the criteria. Relevant clinical data such as clinicopathological characteristics and treatment history were extracted from electronic medical records. All studies were approved by the Dana Farber/Harvard Cancer Center Institutional Review Board (IRB protocol 13-416). Patients provided written informed consent for data collection, blood collection, and downstream analysis. Plasma samples from healthy donors (n=46) were obtained commercially (Innovative Research, 46430 Peary Court, Novi, MI 48377).

**Table 3:** Patient demographics and characteristics.

Characteristic	Patients			
	HER2^+^ N= 40 Number (%)	HER2^-^ N= 39 Number (%)	Total n = 79 Number (%)
Demographic characteristics			
Age (years, m+/-s)	54.5 (+/-12.7)	59.1 (+/-9.6)	56.9 (+/-11.39)
Race			
Black	2 (5%)	1 (2.6%)	3 (3.8%)
Asian	1 (2.5%)	1 (2.6%)	2 (2.5%)
White	33 (82.5%)	36 (92.3%)	69 (87.3%)
Other	3 (7.5%)	0	3 (3.8%)
Not stated	1 (2.5%)	1(2.6%)	2 (2.5%)
Treatment history			
Radiation treatment			
Yes	28 (70.0%)	32 (82.1%)	60 (75.9%)
No	12 (30.0%)	7 (17.9%)	19 (24.1%)
Trastuzumab at blood collection			
Yes	34 (85.0%)	–	–
No	6 (15.0%)	–	–
Other type of therapy at blood col.			
CDK4/6 (single or in combo)	1 (2.5%)	21 (53.8%)	22 (27.8%)
PIK3CA/mTOR (single or in combo)	1 (2.5%)	8 (20.5%)	9 (11.4%)
Chemotherapy	3 (7.5%)	3 (7.7%)	6 (7.6%)
Immunotherapy	0	1 (2.6%)	1 (1.3%)
Targeted therapy	1 (2.5%)	5 (12.8%)	6 (7.6%)
Endocrine therapy (single agent)	0	5 (12.8%)	5 (6.3%)

**Table 4:** Patient clinicopathologic features.

Clinicopathologic features	Patients		
	HER2^+^ N = 40 Number (%)	HER2^-^ N = 39 Number (%)	Total n = 79 Number (%)
ER			
Positive	33 (82.5%)	34 (87.2%)	67 (84.8%)
Negative	7 (17.5%)	5 (12.8%)	12 (15.2%)
PR			
Positive	23 (57.5%)	29 (74.4%)	52 (65.8%)
Negative	17(42.5%)	10 (25.6%)	27 (34.2%)
Metastatic			
Yes	22 (55%)	30 (76.9%)	52 (65.8%)
No	18 (45%)	9 (23.1%)	27 (34.2%)
Histology grade			
Grade 1	–	7 (17.9%)	7 (8.9%)
Grade 2	8 (20.0%)	16 (41.0%)	24 (30.4%)
Grade 2-3	4 (10.0%)	–	4 (5.1%)
Grade 3	23 (57.5%)	11 (28.2%)	34 (43.0%)
Unknown	5 (12.5%)	5 (12.8%)	10 (12.6%)
Invasive histologic type			
Ductal	31 (77.5%)	30 (76.9%)	61 (77.2%)
Lobular	4 (10.0%)	7 (17.9%)	11 (13.9%)
Mixed	5 (12.5%)	2 (5.1%)	7 (8.9%)
Lymph node invasion			
Yes	23 (57.5%)	26 (66.7%)	49 (62.0%)
No	15 (37.5%)	9 (23.1%)	24 (30.4%)
Unknown	2 (5.0%)	4 (10.2%)	6 (7.6%)
Lymphovascular invasion			
Yes	32 (80.0%)	13 (33.3%))	45 (57.0%)
No	6 (15.0%)	22 (56.4%)	28 (35.4%)
Unknown	2 (5.0%)	4 (10.3%)	6 (7.6%)

### Plasma Collection

Blood samples (10 mL) were collected in cfDNA BCT tubes (Streck Inc., La Vista, NE, USA) at an arbitrary time point coinciding with the patients’ clinical visits. Samples were stored ambient for up to 7 days and were centrifuged at 1000 x g for 15 min at 2-8°C. In one instance, plasma was previously isolated from whole blood by double centrifugation at 1,600 x g for 10 min followed by 3,000 x g for 10 min. The resulting plasma was frozen at -80°C, and later thawed for analysis.

### Measurement of cMLC-1 by ELISA

A sandwich ELISA was performed using the human cMLC-1 ELISA kit from MyBioSource (Cat# MBS2506936, San Diego, CA, USA) according to the protocol provided. Each plasma sample was 1:10 diluted with 2% BSA/PBS and then added into anti-cMLC-1 antibody pre-coated wells and incubated at 37°C for 1.5 h. The plasma samples were then decanted. Next, 100 µL of biotinylated anti-cMLC-1 detection antibody working solution was added to each well, and incubated for 1 h at 37°C. After decanting the solution, the wells were washed with the provided washing buffer 3 times. One hundred µL of horseradish peroxidase conjugated avidin (HRP-avidin) working solution was added to each well, and incubated for 30 min at 37°C. The non-bound HRP-avidin was removed by washing with the buffer 5 times. To generate the colorimetric signal, 90 µL of substrate reagent was added to each well. After incubation for 15 min, the enzymatic reaction was stopped with 50 μL stop solution. The optical density (OD) of each well was measured with a microplate reader (Epoch, BioTeck Instrument, Winooski, VT, USA) at a wavelength of 450 nm. In the same test, serial concentrations of standard cMLC-1 working solution (provided in the kit) were included. A four-parameter logistic curve on log-log equation was followed to draw the calibration curve. In each experiment, the standard cMLC-1 protein and all plasma samples were tested in duplicate.

To establish an appropriate detection method, each plasma sample undiluted and at 2-fold dilutions was tested to confirm that its cMLC-1 concentration was within the detectable range of the ELISA kit (0.625 to 40 ng/mL). Based on this titration experiment, a 1:10 dilution of plasma samples was found to be optimal for detection of cMLC-1 concentrations that fit well within the standard curve. The calibration curve following a four-parameter logistic curve on log-log equation was: y = A^2^+ (A_1_-A_2_)/(1+(x/x_0_) p), where A_1 _= 0.051, A_2 _= 6.401, x_0 _= 96.859, p= 0.887, R^2 ^= 0.996. All the plasma samples were tested at least 2 times with a duplicate of each sample, and the concentration of cMLC-1 was calculated according to the calibration equation.

### Statistical Analysis

Paired samples were compared with the paired *t* test. Area under the curve (AUC) was used to evaluate the clinical performance of the tests, and estimates of sensitivity, specificity, and predictive values were calculated and reported with 95% confidence intervals (CI). The medians of foci intensity distributions were tested with the Mann-Whitney U test. One-way ANOVA was used for multiple samples. Data are expressed as mean ± SD of the number of biological replicates indicated in each figure legend. Values of *p* < 0.05 were considered significant.

## Results

### Plasma cMLC-1 Levels of Trastuzumab-Treated Breast Cancer Patients

**Figure 1:**
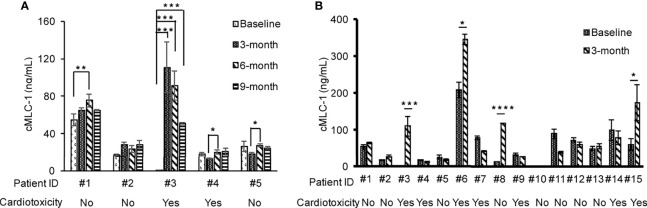
Profile of plasma cMLC-1 levels in trastuzumab-treated breast cancer patients with or without cardiotoxicity. Plasma samples were collected at multiple time points as indicated. Each plasma sample was diluted 1:10 and tested in duplicate to determine cMLC-1 concentration by ELISA. The mean ± SD of cMLC-1 in each sample is shown. Baseline: before trastuzumab treatment; 3, 6 and 9 months: time points after trastuzumab treatment **(A)**. A total of 15 paired-plasma samples collected at before (baseline) and after 3-months trastuzumab treatment were measured for cMLC-1 **(B)**. The mean ± SD of cMLC-1 in each sample is shown. The paired Student -t test was used to analyze the differences. *p<0.05; **p <0.01; ***p <0.005 and ****p <0.001.

To investigate if the association of reduced LVEF and elevated cMLC-1 that we previously noted in mice was clinically relevant in humans, archived plasma samples obtained from breast cancer patients (n=5) administered anthracyclines followed by taxanes and trastuzumab were analyzed for cMLC-1. The archived samples had been collected at multiple time points (baseline, 3, 6, and 9 months). Of the five patients, patients #3 and #4 developed cardiotoxicity. Compared to baseline, cMLC-1 increased in patient #3, but not in patient #4 nor the three patients, #1, #2 and #5, who did not develop TIC (**Figure 1**; **Table 1**). It is worth nothing that, unlike the huge difference of cMLC-1 between 0 and 3 months in patient #3, the changes of cMLC-1 were small between 3 and 6 months in patients #1, #4 and #5. Additionally, there was no change between 3 and 6 months in patient #2, or between 6 and 9 months in any of the 5 patients (**Figure 1A**; **Table 1**). Thus, we decided to test samples from an additional 10 patients (total n=15). These archived plasma samples were collected from these patients at baseline (prior trastuzumab treatment) and at 3 months after initiation of trastuzumab treatment. Five of 10 patients in this cohort developed TIC. Therefore, of the combined total of 15 trastuzumab-treated patients, seven developed TIC, eight did not. The 3-month plasma cMLC-1 measurements were significantly higher than baseline in 3 of the 7 TIC patients, #3, #6 and #15 (3/7, 43%) (**Figure 1B**; **Table 2**). However, only one of the 8 non-TIC patients, #8 (1/8, 13%) had a higher cMLC-1 level at 3 months compared to baseline (**Figure 1B**; **Table 2**). Although this difference was noticeable and indicative, but due to the small sample size, our current available data are not conclusive to support an association between elevated cMLC-1 plasma levels and TIC in trastuzumab-treated breast cancer patients. However, the presented data provide a basis suggesting that the possibility of using plasma cMLC-1 as a biomarker for TIC should be further investigated and validated in a larger cohort of patients with and without cardiotoxicity following trastuzumab treatment.

### Elevated Plasma cMCL-1 Is Associated With Breast Cancer

**Figure 2:**
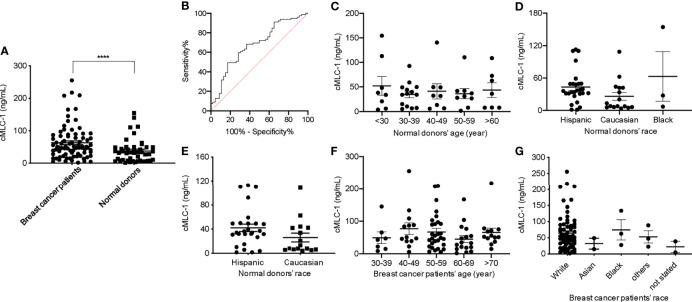
Plasma cMLC-1 level was significantly higher in breast cancer patients than normal healthy women. Each plasma sample was 1:10 diluted and tested twice in duplicate to determine cMLC-1 concentration by ELISA. The mean ± SD of cMLC-1 in breast cancer patients (n=79) *vs.* normal donors (n=46) is shown. The Mann-Whitney U test was used to analyze the difference. ****p=0.0006 **(A)**. The receiver operator characteristic (ROC) graph of the logistic regression result was calculated by GraphPad Prism 8 to determine the relationship between sensitivity and specificity. The cutoff of cMLC-1 at (or higher) 49.55 ng/mL was chosen to reach a sensitivity of 59.49% **(B)**. To determine the impact of age and race factors on cMLC-1 level, plasma samples from all normal donors of different ages and races (n=46) were analyzed and compared. The one-way ANOVA was used for differences among all indicated groups of age (*p*=0.8630) **(C)** and of race (*p*=0.138) **(D)**. To ensure the data were accurate, cMLC-1 between “Hispanic” and “Caucasian” were analyzed by the Mann-Whitney U test (*p*=0.0988) **(E)** without the “black” group given its small size of samples. Plasma samples from all patients of different ages and races (n=79) were analyzed and compared. The one-way ANOVA was used to test differences among all indicated groups of age (*p*=0.4767) **(F)** and of race (*p*=0.7079) **(G)**.

There are currently no blood-based biomarkers approved for the detection of breast cancer. In establishing a reliable assay to measure cMLC-1 protein in plasma, we observed that cMLC-1 levels were significantly higher in samples obtained from breast cancer patients (n=20) than from normal donors (n=10). To validate this finding, cMLC-1 levels in plasma samples obtained from additional 59 patients (total n=79) and 36 normal donors (total n=46) were determined. The final results established that the cMLC-1 level was significantly higher in plasma of patients with breast cancer than in normal donors (63.18 ± 55.31 ng/mL *vs.* 37.61 ± 35.39 ng/mL, *p*=0.0006) (**Figure 2A**). The receiver operator characteristic (ROC) curve analysis of breast cancer (HER2^-^ and HER2^+^) *vs*. normal donors determined area under curve (AUC) value of the logistic regression is 0.6791 (*p*=0.0009). It shows the cutoff cMLC-1 concentration is at 44.99 ng/mL for detecting breast cancer with a sensitivity of 59.49% (95%CI: 48.47%-69.63%) and specificity of 71.74% (95%CI: 57.45% -82.68%) (**Figure 2B**). It is also noteworthy that cMLC-1 did not vary across age or race groups in normal donors (**Figures 2C** **–E**) or in patients (**Figures 2F, G**; **Table 3**). Collectively, this finding suggests cMLC-1 may be a novel potential biomarker combined with other methods and/or biomarkers for breast cancer screening.

### cMCL-1 Plasma Levels in HER2^-^ and HER2^+^ BC Patients

**Figure 3:**
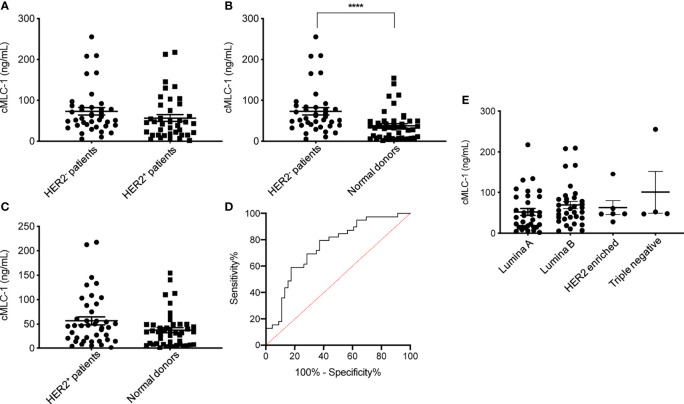
Plasma cMLC-1 levels in HER2^-^, HER2^+^ and different molecule subtypes of breast patients. The mean ± SD of cMLC-1 in HER2^-^ (n=39) *vs.* HER2^+^ (n=40) is shown (*p*=0.0578) **(A)**. Plasma cMLC-1 was much higher in HER2^-^ patients than in normal donors(n=46) (****p < 0.0001) **(B)**; plasma cMLC-1 was noticeable but not significantly higher in HER2^+^ patients than in normal donors (*p*=0.0549) **(C)**. The Mann-Whitney U test was used to analyze the above differences between every two groups. ROC curve analysis determined area under curve (AUC) value of the logistic regression is 0.7480 (*p*<0.0001), indicating cMLC-1 at (or higher) the cutoff of 37.17 ng/mL could predict HER2^-^ breast cancer **(D)**. The means ± SD of cMLC-1 in each subtype of Luminal A (n=35), Luminal B (n=34), HER2 enriched (n=6) and triple negative (n=4) breast cancer patients were analyzed and compared using the one-way ANOVA test (*p*=0.2864) (E).

Next, we analyzed and compared plasma cMCL-1 to determine if it is a potential biomarker for subtyping breast cancer. Interestingly, it was determined that HER2^-^ patients (n=39) had a noticeable, but not significant, increase in their cMLC-1 plasma level compared to HER2^+^ patients (n=40) (73.22 ± 55.88 ng/mL *vs.* 56.67 ± 52.34 ng/mL, *p*=0.0578) (**Figure 3A**). Compared to normal donors, however, HER2^-^ patients had a significant 2.0-fold higher level of cMLC-1 (73.22 ± 55.88 ng/mL *vs.* 37.61 ± 35.39 ng/mL, *p*<0.0001) (**Figure 3B**). As a result, an improved sensitivity of 79.49% (95%CI: 64.47%-89.22%) with the specificity of 63.04% (95%CI:48.60%-75.48%) was for cMLC-1 to predict HER2^-^ breast cancer with the cutoff at 37.17 ng/mL. In line with this finding, cMLC-1 plasma level in HER2^+^ patients was noticeable, but not significant, higher compared to that of normal donors (56.67 ± 52.34 ng/mL *vs.* 37.61 ± 35.39 ng/mL, *p*=0.0549) (**Figure 3C**). ROC curve analysis of HER2^-^ *vs.* normal donors determined AUC value of the logistic regression is 0.7480 (*p*<0.0001) (**Figure 3D**). Subsequently, we compared cMLC-1 levels among the 4 major molecular subtypes of BC patients (n=79): Luminal A (estrogen-receptor (ER)^+^ or progesterone-receptor (PR)^+^, HER2^-^, n=35), Luminal B (ER^+^ or PR^+^, HER2^+^, n=34), HER2-enriched (ER^-^, PR^-^, HER2^+^, n=6) and triple negative breast cancer (TNBC) (ER^-^, PR^-^ and HER2^-^, n=4) (20, 21). It was found no significant difference among the 4 subtypes (**Figure 3E**). However, we should interpret the results with caution since the sample sizes in HER2-enriched and TNBC are too small, which reduces the power of the study, to get a conclusive statistical analysis.

### Plasma cMCL-1 Is a Potential Biomarker for Breast Cancer Progression

**Figure 4:**
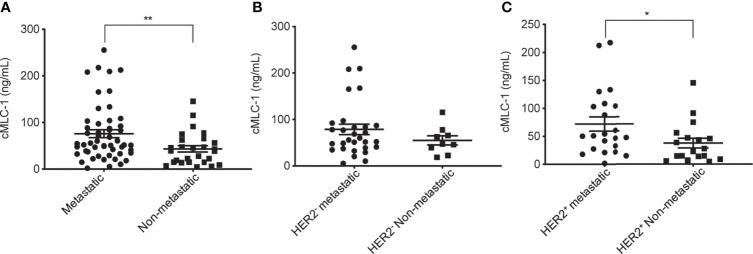
Plasma cMCL-1 was higher in metastatic than non-metastatic breast cancer patients. The means ± SD of cMLC-1 in metastatic (n=52) *vs.* non-metastatic patients (n=27) are shown (**p=0.0069) **(A)**. Plasma cMLC-1 was not significantly different in HER2^-^ metastatic (n=30) *vs.* non-metastatic patients (n=9) (*p*=0.3657) **(B)**. Plasma cMLC-1 was higher in HER2^+^ metastatic (n=22) *vs.* non-metastatic patients (n=18) (*p=0.0204) **(C)**. The Mann-Whitney U test was used to analyze the above differences between every two groups.

We then wanted to assess if cMCL-1 levels differ in patients with or without metastasis. As shown in **Table 4**, 52 out of 79 patients (65.8%) analyzed had metastatic disease. We found that cMLC-1 level was higher in patients with metastatic breast cancer than in patients with early or locally advanced breast cancer, or non-metastatic breast cancer (75.96 ± 59.85 ng/mL *vs.* 43.41 ± 34.26 ng/mL, *p=*0.0072) (**Figure 4A**). Although it is highly unlikely, this finding may be somewhat affected by the fact that more metastatic BC patients (30/52, 57.7%) were HER2^-^ with high cMLC-1 levels, while less metastatic BC patients (22/52, 42.3%) were HER2^+^ with comparatively lower cMLC-1 levels (**Table 4** **).** No significant difference was noted in the cMLC-1 plasma level of metastatic (n=30) versus non-metastatic (n=9) HER2^-^ patients (78.68 ± 60.78 ng/mL *vs.* 54.99 ± 28.54 ng/mL, *p*=0.3657) (**Figure 4B**), while HER2^+^ patients with metastatic disease (n=22) had a significantly higher level of cMLC-1 than non-metastatic HER2^+^ patients (n=18) (72.26 ± 58.37 ng/mL *vs.* 37.62 ± 35.57 ng/mL, *p*=0.0204) (**Figure 4C**). Our results indicate that the cMLC-1 level may be associated with the progression of disease and may serve as a potential biomarker for metastasis as well as monitoring response to therapy.

### Plasma cMCL-1 Level and Additional Clinicopathological Characteristics of Patients

**Figure 5:**
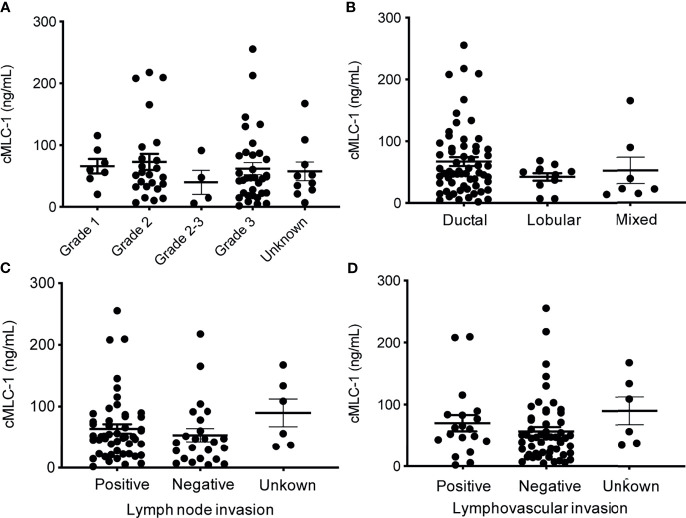
Plasma cMCL-1 levels in breast cancer patient sub-cohorts divided by various clinicopathological features. The means ± SD of plasma cMLC-1 levels in groups of histology grade [Grade 1 (n=7), Grade 2 (n=24), Grade 2-3 (n=4), Grade 3 (n=34), unknown (n=10)] (*p*=0.8233) **(A)**; in ductal (n=61), lobular (n=11), and mixed histology characters (n=7) (*p*= 0.3169) **(B)** are shown. The means ± SD of cMLC-1 levels in patients with lymph node (+) (n=49), lymph node (–) (n=24), unknown (n=6) (*p*=0.3163) **(C)**; and in patients with lymphovascular invasion (+) (n=19), without (–) (n=54), unknown (n=6) (*p*=0.2730) **(D)** are shown. The one-way ANOVA was used to test differences among all indicated multiple groups.

Next, we investigated if cMCL-1 level was associated with additional clinicopathological characteristics collected from patients at the time of diagnosis/treatment. First, plasma cMCL-1 level was compared among 69/79 patients with known histologic grade 1 (n=7), 2 (n=24), 2-3 (n=4) or 3 (n=34), and no difference was found (**Figure 5A**; **Table 4**). Second, plasma cMCL-1 level was compared among cohorts of patients with different histologic types, i.e., ductal (n=61), lobular (n=11), and mixed (n=7), and no difference was found (**Figure 5B**; **Table 4**). Third, plasma cMCL-1 level was compared between known lymph node positive (n=49) *vs.* negative (n=24) cohorts and no difference was found (63.54 ± 52.48 ng/mL *vs.* 53.23 ± 50.64 ng/mL, *p*=0.3163) (**Figure 5C**; **Table 4**). Lastly, plasma cMCL-1 level was compared between patients with known lymphovascular invasion (LVI) (n=19) *vs.* without LVI (n= 54) and no difference was found (70.08 ± 55.48 ng/mL *vs.* 56.65 ± 50.41 ng/mL, *p*=0.2730) (**Figure 5D**; **Table 4**).

### Plasma cMCL-1 Levels Are Stable at -80°C Over Time

**Figure 6:**
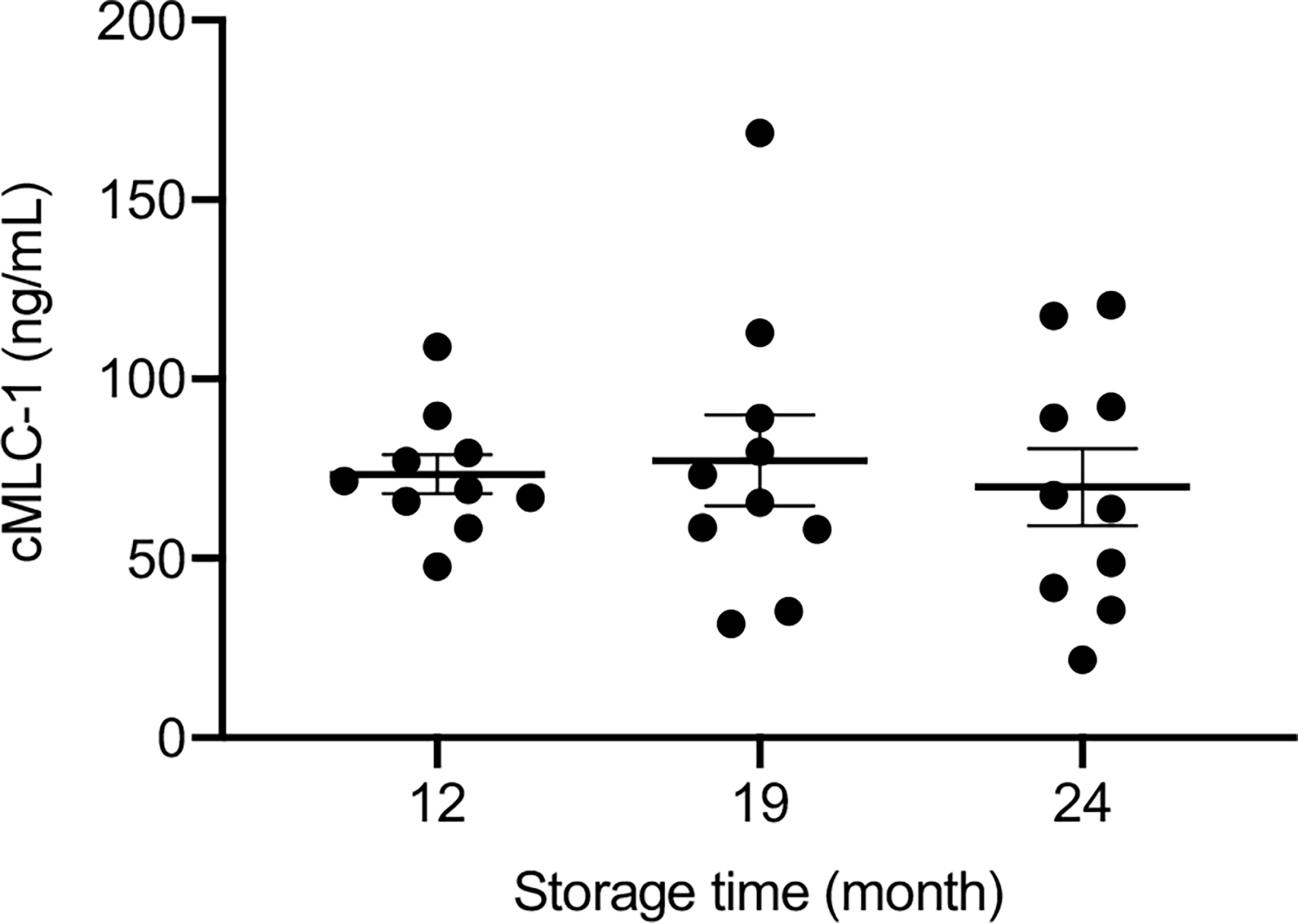
Stability of cMCL-1 in plasma stored at -80°C. Plasma cMLC-1 levels were repeatedly tested in samples from breast cancer patients and normal donors. Representative data using the same set of normal donors samples (n=10) stored at -80°C for 12, 19 and 24 months are shown. The one-way ANOVA was used to test the difference (*p*=0.8737).

To evaluate the stability of cMLC-1 in archived plasma samples over time, plasma samples kept at -80°C storage for 12, 19 and 24 months were analyzed for this marker. The levels of cMCL-1 were consistent across all time points tested for specimens from a given breast cancer patient or normal donor. Representative data are shown (**Figure 6**), and indicate that cMLC-1 in plasma samples stored at -80°C is stable for at least 2 years. Its long-term stability will facilitate its use as a biomarker and in other clinical studies.

## Discussion

The initial results of this research indicated that cMLC-1 plasma levels were elevated with respect to baseline in 4/15 trastuzumab treated breast cancer patients studied. Of these four patients, three did but one did not develop TIC. Due to its small sample size, however, this analysis did not validate cMLC-1 as a biomarker for early detection and prediction of TIC. Consequently, the current data does not support our hypothesis that elevated cMCL-1 plasma levels are indicative of TIC. Nevertheless, our data suggests it is worthy to further test this hypothesis employing a larger cohort of patients to assess its clinical significance. For future studies, sample collection should be timed within the 3 month period (i.e. 1-week, 2-week, 1-, 2- and 3- months post-treatment with trastuzumab) in order to validate cMLC-1 as a biomarker for early detection or prediction of TIC (16, 17). It is also worthy to point out that plasma cMLC-1 may serve as a cardiotoxicity marker but not specifically for TIC based on previous study results (16–18).

To date, the most effective means of early detection and screening for breast cancer involves mammography, a technique that has been approved and widely practiced since the 1980s (22). Though mammography has undoubtedly improved outcomes for women with breast cancer—research estimates at least a 50% mortality reduction since becoming standard practice—this method is imperfect and presents its own challenges (23). Yearly mammograms are recommended starting at age 40, which is often too late for women with some of the more aggressive forms of breast cancer. Additionally, due to the nature of the exam and the frequency at which it is required, attendance rates among women for their yearly mammograms vary, suggesting an additional layer of more accessible screening measures may help close the gap (24). Finally, the false positive rate for mammography is alarmingly high. In the U.S., the 10-year false positive rate is 30%, and 50% of all women will receive a false positive result at some point (24). The ideal solution for breast cancer screening is a blood-based biomarker that can complement or replace the flawed practice of mammography to overcome its shortcomings. A blood test is generally far easier to schedule, better tolerated by patients, and can be integrated into a routine clinical visit. Additionally, a blood test can be justifiably introduced earlier than age 40, as it will be less expensive for payers.

Currently, no blood biomarkers for breast cancer diagnosis or screening have been approved for clinical use. Our data suggest that elevated cMLC-1 (79 patients consisting of ~50% each of HER2^-^ and HER2^+^) *vs.* 46 normal donors, *p*=0,0006) may be a potential candidate as a biomarker for initial and/or combinational screening of women under 40 who are at high risk for breast cancer (**Figure 2**). It is noteworthy that plasma cMCL-1 might be more sensitive in predicting HER2^-^ breast cancer as cMLC-1 is noticeably but not significantly higher in HER2^-^ than HER2^+^ patients (**Figure 3**). However, since cMLC-1 levels are higher in both HER2^-^ (*p*<0.0001) and HER2^+^ patients (*p*=0.0549) compared to normal donors, although the latter difference did not reach a statistically significant level with the given small sample size, we anticipate that a larger sample size would result in a narrower 95% confidence interval for sensitivity and specificity to predict breast cancer, regardless of HER2 status.

Importantly, plasma cMLC-1 levels are correlated with disease progression; it is higher in metastatic (n=52) than in non-metastatic (n=27) breast cancer patients (75.96 ± 59.85 ng/mL *vs.* 43.41 ± 34.26 ng/mL, *p=*0.0072) (**Figure 4A**). It is also noticeable that a lack of significant difference was found in the cMLC-1 plasma level of metastatic (n=30) versus non-metastatic (n=9) HER2^-^ patients (*p*=0.3657) (**Figure 4B**), while a significantly higher level of cMLC-1 was found in metastatic (n=22) than non-metastatic (n=18) HER2^+^ patients (*p*=0.0237) (**Figure 4C**). The data on cMCL-1 level in HER2^-^ patients in relation to metastasis must be explained with caution since the sample size of non-metastatic HER2^-^ patients is small (n=9). In contrast, the data on cMCL-1 level in HER2^+^ patients in relation to metastasis seems to be more convincing due to bigger sample sizes from both cohorts used for the statistical analysis. In addition, no association of plasma cMCL-1 was established with either any of the 4 major molecular subtypes of breast cancer or any of the additional clinicopathological characteristics of patients analyzed, including age, race, histologic grade, invasive histologic types, breast cancer spread to lymph node (+ *vs.* -) or LVI (+ *vs.* -) (**Figure 5**). Again, these preliminary data must be interpreted cautiously since the small number of plasma samples from each sub-cohort was examined. This is the limitation of this study. Despite the limitation, our overall data on elevated plasma cMCL-1 found in breast cancer patients vs. normal donors as well as in metastatic vs. non-metastatic breast cancer patients provided valuable information on the first attempt to assess cMCL-1 as a potential biomarker for disease screening and therapy monitoring. The method described here to detect plasma cMLC-1 is fast and easy. Moreover, we demonstrated that plasma cMLC-1 is stable over time after storing in -80°C freezers for at least 2 years (**Figure 6**). We acknowledge that the sample sizes are seemed small for our sub-aim studies between cohorts, i.e., HER2^-^ (n=39) *vs.* HER2^+^ (n=40), metastatic (n=52) *vs.* non-metastatic (n=27). However, the aim of these additional analyses was not to identify and validate plasma cMLC-1 as an ultimate biomarker but to explore the potential for a promising biomarker to decide whether an enlarged study is worthwhile to pursue (25). Therefore, besides having presented our statistical analysis-based conclusions, here we provided all detailed information regarding the technical and statistical analysis methods for our colleagues who may have a large number of archived patient samples to validate our study results timely. We believe our initial data as they stand now would serve as a first stepping stone and new idea to facilitate and attract more studies from the research field to evaluate the potential of cMLC-1 as a biomarker for breast cancer screening and disease progression.

## Conclusion

The results of this investigation provide a sound basis for the novel and exciting further investigation of cMLC-1 as a blood protein biomarker for screening breast cancer, evaluating disease progression, monitoring treatment response and predicting TIC. Furthermore, our study highlights the need to define the mechanisms(s) of how and why plasma cMLC-1 is elevated in breast cancer patients.

## Data Availability Statement

The raw data supporting the conclusions of this article will be made available by the authors, without undue reservation.

## Ethics Statement

All studies were approved by the Massachusetts General Hospital Institutional Review Board (IRB protocol 2006P000886) and the Dana Farber/Harvard Cancer Center Institutional Review Board (IRB protocol 13-416). Patients provided written informed consent for data collection, blood collection, and downstream analysis. The patients/participants provided their written informed consent to participate in this study. Written informed consent was obtained from the individual(s) for the publication of any potentially identifiable images or data included in this article.

## Author Contributions

XW, WW, SI, and MS-C conceived the study and designed the experiments. LJ, LY, and TS carried out the experiments. RA, AK, CW, KH, and AV collected patient sample collection and provided de-identified patient information. CP and DM supported the patient sample collection. RA, AK, and LJ analyzed and organized patient information. LY, RA, LJ, HZ, and AL analyzed the data. XW, LY, and RA interpreted the data. XW, LY, RA, and HZ wrote the manuscript. All the authors read and approved the submitted manuscript.

## Funding

This work was supported by grants 1R30FD006290-01 U.S. Food & Drug Administration (XW), R01CA226981-01A1 (XW), the FDA Office of Women’s Health Research Science Program Award (Project ID: 750912CDR, WW), China scholarship council (No. 201906995004, LY), Susan G. Komen for the Cure (SCM) and NIH/NHLBI R01HL130539 (SCM).

## Conflict of Interest

The authors declare that the research was conducted in the absence of any commercial or financial relationships that could be construed as a potential conflict of interest.

## Publisher’s Note

All claims expressed in this article are solely those of the authors and do not necessarily represent those of their affiliated organizations, or those of the publisher, the editors and the reviewers. Any product that may be evaluated in this article, or claim that may be made by its manufacturer, is not guaranteed or endorsed by the publisher.

## Abbreviations

AUC, area under the curve; BC, breast cancer; BNP, brain natriuretic peptide; CEA, carcinoembryonic antigen; CI, confidence intervals; cMLC-1, cardiac myosin light chain 1; CK, creatine kinase; CREC, Cardiac Review and Evaluation Committee for Trastuzumab; ELC, essential light chain; ELISA, enzyme-linked immunosorbent assay; ER, estrogen-receptor; FISH, Fluorescent *In Situ* Hybridization; HER2, epidermal growth factor receptor 2; HRP, horseradish peroxidase; hs-CRP, high-sensitivity C-reactive protein; IHC, immunohistochemistry; LVEF, left ventricular ejection fraction; NT-proBNP, N-terminal pro b-type natriuretic peptide; OD, optical density; PR, progesterone-receptor; ROC, receiver operator characteristic; SD, standard deviation; TIC, Trastuzumab-induced cardiotoxicity; TnI, Troponin-I.
